# STARD9 and CDK5RAP2—Novel Candidate Genes for 46,XY Complete Gonadal Dysgenesis

**DOI:** 10.3390/ijms262311575

**Published:** 2025-11-28

**Authors:** Dmytro Sirokha, Alexey Rayevsky, Vitalii Kalynovskyi, Mykola Khalangot, Oksana Samson, Olexandra Gorodna, Krystyna Kwiatkowska, Chloe Mayere, Zaneta Lemanska, Amanda Kunik, Serge Nef, Kamila Kusz-Zamelczyk, Ludmila Livshits

**Affiliations:** 1 Department of Molecular Genetics, Institute of Molecular Biology and Genetics, National Academy of Sciences of Ukraine, 03143 Kyiv, Ukrainea.v.gorodna@imbg.org.ua (O.G.); 2 Institute of Food Biotechnology and Genomics, National Academy of Sciences of Ukraine, 04123 Kyiv, Ukraine; 3 Department of Molecular Modeling, Enamine Ltd., 02094 Kyiv, Ukraine; 4 Educational and Scientific Centre “Institute of Biology and Medicine”, Taras Shevchenko National University of Kyiv, 03022 Kyiv, Ukraine; vitalii.kalynovskyi@gmail.com; 5 Endocrine Department, Shupyk National Healthcare University of Ukraine, 04112 Kyiv, Ukraine; nikhalangot@ukr.net (M.K.); samson1oksana@gmail.com (O.S.); 6 Institute of Human Genetics, Polish Academy of Sciences, 61-479 Poznan, Poland; krystyna.kwiatkowska@igcz.poznan.pl (K.K.); zaneta.lemanska@igcz.poznan.pl (Z.L.); amanda.kunik@igcz.poznan.pl (A.K.); 7 Department of Genetic Medicine and Development, Faculty of Medicine, University of Geneva Medical School, 1206 Geneva, Switzerland; chloe.mayere@unige.ch (C.M.); serge.nef@unige.ch (S.N.)

**Keywords:** 46,XY gonadal dysgenesis, oligogenic inheritance, STARD9, CDK5RAP2

## Abstract

46,XY gonadal dysgenesis, characterised by absent or defective testicular development in individuals with a 46,XY karyotype, results from disruptions in the genetic programme governing testis determination and differentiation during embryogenesis. While monogenic causes explain approximately 50% of cases, emerging evidence suggests an oligogenic basis in some patients. However, many cases remain without a definitive molecular diagnosis. In this study, we investigated a patient with 46,XY gonadal dysgenesis to explore the underlying genetic aetiology. Whole-exome sequencing in the patient did not reveal any pathogenic variants in genes previously associated with this condition. Instead, it detected rare variants in *STARD9* and *CDK5RAP2*, which encode centrosomal proteins known to interact with each other. Gene expression analysis of embryonic gonads revealed that *STARD9* is sexually dimorphic, with the highest expression in testis-specific Sertoli cells, while *CDK5RAP2* is ubiquitously expressed, including in Sertoli cells. These findings suggest a role for both genes in Sertoli cell development, implicating them in the pathogenesis of 46,XY gonadal dysgenesis. To evaluate the functional relevance of the identified variants, we performed molecular dynamics simulations, which suggest that these variants may impair the individual and/or combined functions of STARD9 and CDK5RAP2 proteins. This study is the first to propose a role for *STARD9* and *CDK5RAP2* genes in human Sertoli cell development and highlights their potential contribution to 46,XY gonadal dysgenesis.

## 1. Introduction

Sexual development in human embryos relies on the correct determination, differentiation, and function of the gonads. Disruptions in these processes—often caused by genetic mutations—result in Disorders/Differences of Sex Development (DSDs), a heterogeneous group of congenital conditions in which chromosomal, gonadal, or anatomical sex has been compromised [1]. Among these conditions is 46,XY gonadal dysgenesis, which encompasses both complete (46,XY CGD) and partial (46,XY PGD) forms. 46,XY CGD is characterised by the presence of streak gonads composed of fibrous tissue lacking testicular differentiation, leading to female-appearing internal and external genitalia despite a 46,XY karyotype. In turn, 46,XY PGD involves atypical gonadal development resulting in varying degrees of genital ambiguity in individuals with a 46,XY karyotype. While many cases of 46,XY gonadal dysgenesis have a monogenic basis with mutation in one of multiple genes essential for testis determination (for a review, refer to [2,3]), emerging evidence suggests that some cases may follow an oligogenic model in which multiple genetic variants collectively contribute to the phenotype [4,5,6]. Notably, approximately 50% of all 46,XY gonadal dysgenesis cases remain unexplained [2], highlighting the need to identify additional genetic factors. Recent large-scale high-throughput sequencing studies continue to reveal new candidate genes associated with 46,XY gonadal dysgenesis.

The aim of this study was to identify novel candidate gene(s) for 46,XY gonadal dysgenesis by analysing the exome of a patient with 46,XY CGD in whom whole-exome sequencing (WES) revealed no mutations in known 46,XY gonadal dysgenesis-associated genes. Based on our findings, we hypothesise that variants in two genes—*Steroidogenic Acute Regulatory-Related Lipid Transfer Domain 9* (*STARD9*) and *CDK5 Regulatory Subunit-Associated Protein 2* (*CDK5RAP2*)—which are expressed in early embryonic Sertoli cells and encode interacting proteins, may act together to impair Sertoli cell development and consequently disrupt testis formation.

## 2. Results

### 2.1. The 46,XY Complete Gonadal Dysgenesis Patient

A 17-year-old female patient was referred to a gynaecologist due to the absence of secondary sexual characteristics. Clinical assessment showed breast development B I-II and pubic hair development PH IV according to Tanner scale. Pelvic ultrasonography revealed a 22 mm × 13 mm × 25 mm uterus, a 17 mm × 12 mm right gonad, and an 18mm × 13 mm left gonad located intra-abdominally. No pelvic magnetic resonance imaging was performed. Hormonal investigations revealed oestradiol (E2) at 31.94 pmol/L, progesterone (P4) at 1.65 nmol/L, and testosterone (T) at 0.7628 nmol/L, near the low level of the reference interval, while gonadotropin levels were markedly elevated: follicle-stimulating hormone (FSH) at 153.2 IU/L and luteinizing hormone (LH) at 41.8 IU/L, consistent with primary (hypergonadotropic) hypogonadism. Cytogenetic analysis revealed a 46,XY karyotype with the presence of an *SRY* gene and no mosaicism. Based on the presence of female external genitalia and uterus, small gonads, primary amenorrhea, elevated gonadotropins, and a 46,XY karyotype, the diagnosis of 46,XY CGD was made.

**Figure 1:**
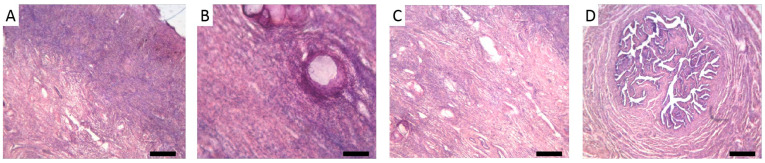
The histology of the patient’s gonad, stained with haematoxylin and eosin. (**A**) The gonad (only one gonad was documented) formed by fibrous connective tissue. The cortical layer of the gonad is denser than the medullary layer. (**B**) The cortical layer with single follicular-like structures that contain detached epithelial tissue and a homogeneous, faintly stained basophilic substance. (**C**) The medullary layer with numerous blood vessels and lacunae. (**D**) Oviduct. Scale bars: 250 µm (**A**,**C**,**D**); 100 µm (**B**).

At 17 years and 4 months of age, the patient underwent laparoscopic gonadectomy, which revealed whitish gonads at the ends of the rudimentary fallopian tubes. Both the gonads and fallopian tubes were excised. Histological analysis confirmed complete gonadal dysgenesis, with the gonads composed of fibrous connective tissue and with no differentiated structures observed (Figure 1). The cortical and medullary layers were distinguishable, with the cortical layer containing single follicular structures (Figure 1A,B), while the medullary layer was characterised by numerous blood vessels and lacunae (Figure 1C). Additionally, a thickened fallopian tube with a highly branched lumen was observed (Figure 1D). Following sex hormone replacement therapy, the patient experienced regular menstrual cycles.

### 2.2. WES Outcome

To identify the genetic cause of 46,XY CGD in the patient, WES trio was performed. The sequencing achieved a mean coverage of >145 reads, with 95.3% of the targets covered at depth of >30 reads. Initial analysis revealed 1,360,189 single-nucleotide variants or small insertion–deletion variants in the patient. After filtering based on quality and minor allele frequency (MAF) < 0.01, a subset of 509 coding sequence variants was retained for further analysis.

### 2.3. Variants in Known DSD Genes

We first screened these 509 variants against a curated list of 198 DSD-associated genes, encompassing both established and candidate genes (Supplementary Materials: List of DSD genes) [7,8,9,10,11,12,13,14,15,16,17]. No pathogenic variants were identified in genes previously linked to 46,XY gonadal dysgenesis. However, we detected three heterozygous variants in autosomal DSD genes not previously associated with 46,XY gonadal dysgenesis: *Chromodomain Helicase DNA Binding Protein 7* (*CHD7*) NM_017780.4:c.2273G>A (p.Arg758His) (rs202208393), *KISS1 Receptor* (*KISS1R*) NM_032551.5:c.1167C>A (p.Cys389Ter) (rs371771794), and *Leucine Rich Repeat Containing G Protein-Coupled Receptor 5* (*LGR5*) NM_003667.4:c.2341C>G (p.Pro781Ala) (rs113809442). Segregation analysis showed that all three variants were inherited from the unaffected father, suggesting that these variants alone are unlikely to cause 46,XY CGD. Nonetheless, their potential modifying effect on the patient’s phenotype cannot be excluded.

### 2.4. Variants in STARD9 and CDK5RAP2 Candidate Genes

Since no convincing causative variants were identified in known DSD-related genes, we analysed the remaining genes harbouring rare variants in the patient. Selection of candidate genes was based on the following criteria: (1) non-synonymous variants; and (2) evidence from the literature suggesting a potential role in gonadal development. This approach yielded *STARD9* and *CDK5RAP2* candidate genes. *STARD9* encodes a protein belonging to the StAR-related lipid transfer (START) domain family, members of which have been implicated in 46,XY gonadal dysgenesis [13,15,18]. *CDK5RAP2*’s mouse homologue, in turn, has been shown to play a role in Sertoli cell polarisation [19]. Notably, STARD9 and CDK5RAP2 proteins localise to the same subcellular structure—the pericentriolar material (PCM) of the centrosome [20,21]—and have been demonstrated to physically interact in a yeast two-hybrid assay [22]. Based on this evidence, we considered a possible cooperative contribution of the *STARD9* and *CDK5RAP2* variants to the 46,XY CGD phenotype.

#### 2.4.1. STARD9 Variants in a Compound Heterozygous State

**Figure 2:**
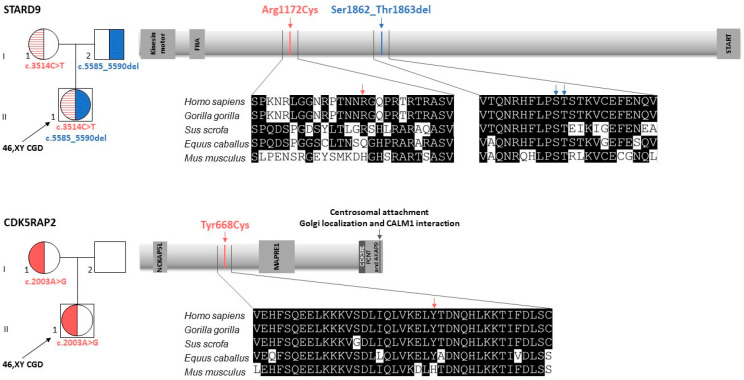
*STARD9* and *CDK5RAP2* variants identified in a patient with 46,XY complete gonadal dysgenesis (46,XY CGD). The inheritance pattern of identified variants is shown on the **left** side of each panel. A schematic representation of STARD9 and CDK5RAP2 proteins, highlighting functional domains and regions critical for protein–protein interactions, as well as the localisation of variants identified in the patient (**top**-**right** section of each panel). Alignments of mammalian STARD9 and CDK5RAP2 fragments with arrows indicating mutation sites at the protein level (**bottom**-**right** section of each panel).

WES identified two *STARD9* variants in a compound heterozygous state in the proband. One was an in-frame deletion, NM_020759.3:c.5585_5590del (p.Ser1862_Thr1863del) (rs528276071), inherited from the heterozygous father. The second one was a missense mutation, NM_020759.3:c.3514C>T (p.Arg1172Cys) (rs12594837), inherited from the heterozygous mother. Both variants were confirmed by Sanger sequencing in the patient and her parents (Supplementary Materials: Figure S1). The MAF of the paternal p.Ser1862_Thr1863del variant is 0.001325, while the MAF of the maternal p.Arg1172Cys variant is 0.009863. Neither variant has been previously described as linked to any disease. Both lie outside functional domains of STARD9. While the Ser1862 and Thr1863 residues are conserved among mammals, the Arg1172 residue is not (Figure 2).

#### 2.4.2. CDK5RAP2 Heterozygous Variant Inherited from the Mother

WES identified a heterozygous missense variant in the *CDK5RAP2* gene in the proband. The identified variant, NM_018249.5:c.2003A>G (p.Tyr668Cys) (rs137966123), was inherited from the heterozygous mother (Figure 2). Sanger sequencing validated the variant in both the patient and her mother (Figure S1). The MAF of the variant is 0.0003284. The variant has not been previously described as implicated in the pathogenesis of any disease. The Tyr668 residue is not conserved and is located outside regions known to be critical for protein localisation or interactions (Figure 2). However, the CDK5RAP2 region required for interaction with STARD9 has not been characterised to date.

### 2.5. STARD9 and CDK5RAP2 Expression in Embryonic Sertoli Cells

**Figure 3:**
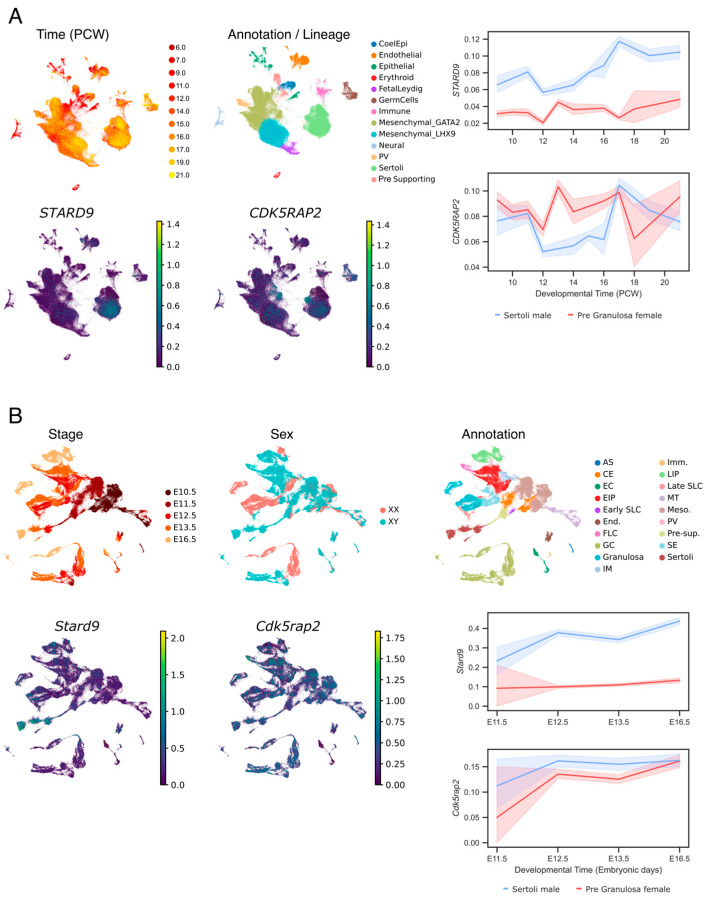
*STARD9* and *CDK5RAP2* expression profile in human (**A**) and mouse (**B**) embryonic gonads based on single-cell transcriptomics. (**A**). Left panel: Uniform Manifold Approximation and Projection (UMAP) visualisation of cells from human male embryonic gonads, coloured by developmental stage, cell annotation, and expression levels of the *STARD9* and *CDK5RAP2* genes. Right panel: Line plot showing the log-scaled expression levels of *STARD9* and *CDK5RAP2* in supporting cells from human embryonic female and male gonads across developmental time points measured in post-conception weeks (PCW). This figure is based on data from Garcia-Alonso et al., 2022 [25]. (**B**). UMAP visualisation of 94,705 cells from mouse embryonic gonads coloured by developmental stage, sex, cell cluster annotations, and expression levels of the *Stard9* and *Cdk5rap2* genes. Cell cluster annotations include: AS, adrenosympathic cells; CE, coelomic epithelial cells; EC, erythrocytes; EIP, early interstitial progenitors; End., endothelial cells; FLC, foetal Leydig cells; GC, germ cells; Granulosa, pregranulosa cells; IM, invading mesonephric cells; Imm., immune cells; LIP, late interstitial progenitors; MT, mesonephric tubules; Meso., mesonephric mesenchymal cells; PV, perivascular cells; Pre-sup., presupporting cells; SE, surface epithelial cells; Sertoli, Sertoli cells; SLC, supporting-like cells. Right-bottom panel: Line plot showing the log-scaled expression levels of *Stard9* and *Cdk5rap2* in supporting cells of embryonic XX and XY mouse gonads across developmental time points measured in embryonic days (E). This figure is based on data from Mayere et al., 2022 [26].

To assess whether *STARD9* and *CDK5RAP2* may play a role in testicular development, we analysed their expression in published single-cell RNA sequencing atlases of human and mouse embryonic gonads. According to Uniform Manifold Approximation and Projection (UMAP) and line plot visualisations, at mouse embryonic day 11.5, when gonadal sex determination takes place, *Stard9* expression was higher in Sertoli cells than in granulosa cells. Subsequently, *Stard9* expression further increased in Sertoli cells and remained significantly higher than in granulosa cells (Figure 3B). In humans, *STARD9* expression was also significantly higher in Sertoli cells compared with granulosa cells (Figure 3A). These findings show that *STARD9*/*Stard9* exhibits sexually dimorphic expression, with the highest levels observed in male-specific Sertoli cells. Together, these findings suggest an evolutionarily conserved role for *STARD9*/*Stard9* in Sertoli cell development. In turn, *CDK5RAP2*/*Cdk5rap2* exhibited ubiquitous expression across embryonic gonadal cell types; notably, this gene is expressed in Sertoli cells at the same developmental stages as *STARD9*/*Stard9* (Figure 3). This co-expression pattern suggests that *STARD9*/*Stard9* and *CDK5RAP2*/*Cdk5rap2* may act cooperatively in Sertoli cell development, implicating them in the pathogenesis of 46,XY gonadal dysgenesis.

### 2.6. Investigating the Functional Consequences of STARD9 and CDK5RAP2 Variants

#### 2.6.1. Both STARD9 p.Arg1172Cys and p.Ser1862_Thr1863del Variants Reside in Intrinsically Disordered Regions

**Figure 4:**
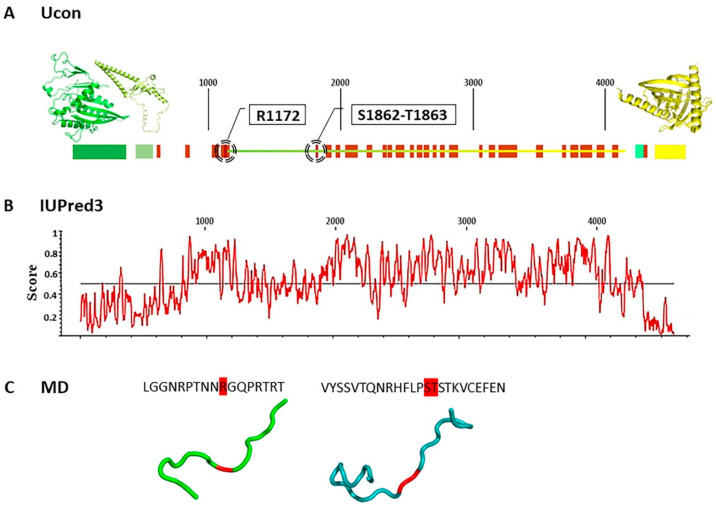
Workflow of STARD9 protein structure prediction. (**A**) Secondary structure prediction of the full-length STARD9 protein obtained using the Ucon predictor and homology modelling to kinesin-3 KLP-6. The prediction confirmed the presence of structured domains: kinesin motor (dark green), FHA (light green), and START (yellow), and revealed many disordered regions (red). Both studied STARD9 variant sites are located within predicted disordered regions. (**B**) Secondary structure prediction of the full-length STARD9 protein, obtained using IUPred3 predictor based on the amino acid sequence and energy estimation approach, reflecting the probability of the ordered regions (located above the median line) and disordered regions (located below the median line). It predicts the region containing Arg1172 to be ordered, whereas Ser1862_Thr1863 to be disordered. (**C**) The most frequent conformations of the short STARD9 fragments containing either the p.Arg1172Cys or p.Ser1862_Thr1863del mutation, obtained using molecular dynamics, showing the disordered structure of the studied fragments. The red colored background for the sequence was used to highlight the residues affected by mutation and point the position in the peptide.

To assess the potential impact of STARD9 p.Arg1172Cys and p.Ser1862_Thr1863del variants on protein structure, we applied in silico approaches, as the STARD9 protein has not yet been crystallised. Both variants are located in the central region of the protein, outside functional domains. We focused on analysing this central segment to predict how these variants might alter STARD9 structure. To this end, we first predicted the secondary structure of full-length STARD9. Most of the predictors we applied indicated that Arg1172 and Ser1862_Thr1863 are located in an unstructured region (Figure 4A), whereas some analyses suggested that Ser1862_Thr1863 residues could be involved in the formation of an ordered region (Figure 4B). AlphaFold 3D structure predictions further support that these sequences are part of an intrinsically disordered region (IDR), consistent with their amino acid composition.

To validate the secondary structure predictions, we performed 3D reconstruction and molecular dynamics (MD) simulations. Coarse-grain MD simulations were conducted on the wild-type STARD9 core fragment containing Arg1172 and Ser1862_Thr1863, to evaluate the behaviour in solution and assess the probable effect of these mutations. However, due to the mosaic-like structure of this protein fragment, which contained a mix of unstructured regions and secondary structure elements, we did not obtain significant results that could provide an exhaustive description of its behaviour. The fragment was highly unstable, preventing its use as a template structure for mutation analysis.

To address the limitations of coarse-grain MD simulations, we performed full-atom MD simulations of mutant fragments, each containing one of the identified mutations (p.Arg1172Cys or p.Ser1862_Thr1863del). These simulations aimed to model the secondary structure folding process directly on the mutated sequences. Results revealed that both fragments retained their disordered nature, showing no tendency to form secondary structures (Figure 4C). These results, together with the secondary structure predictions and AlphaFold analysis, provide consistent premises that both p.Arg1172Cys and p.Ser1862_Thr1863del variants reside within IDR. Predicting functional consequences in IDRs remains a significant challenge in computational biology, but it is important to note that IDRs often mediate protein–protein interactions [23,24], and alterations in their sequence can potentially affect binding properties. However, confirming any such effect for the STARD9 variants studied here would require experimental approaches.

#### 2.6.2. CDK5RAP2 p.Tyr668Cys Variant Lies Within the Alpha-Helix

To assess the potential structural impact of the p.Tyr668Cys variant in CDK5RAP2, we conducted in silico modelling using AlphaFold 2, which generated multiple structural models characterised by 9 to 14 α-helices. A central α-helix-based scaffold was a common feature across all models, forming a structural backbone of the protein. However, the smaller helices, connected by flexible linkers, exhibited variability in their spatial arrangement.

Importantly, in all models, the p.Tyr668Cys CDK5RAP2 variant was located in a central region of the α-helix (residues ~650–684). Our 200 ns MD simulation of both the intact and mutant helices indicated a stable secondary structure in both cases. However, based on structures with similar substitutions [27,28], this mutation may influence inter-domain interactions and thus may affect CDK5RAP2 function.

## 3. Discussion

In this study, we investigated a case of 46,XY CGD and performed WES trio to explore the genetic basis of gonadal dysgenesis in the patient. No pathogenic variants were identified in genes previously associated with 46,XY gonadal dysgenesis. Instead, we discovered rare variants in *STARD9* and *CDK5RAP2,* and we consider the possibility that these variants together might contribute to the phenotype.

*STARD9* and *CDK5RAP2* encode proteins that physically interact, as demonstrated by the yeast two-hybrid assay [22], and both localise to the PCM of the centrosome [20,21]. STARD9 has been shown to be essential for PCM stabilisation, and its disruption leads to spindle fragmentation, apoptosis, impaired microtubule dynamics, and abnormal chromosome segregation [21,29], while CDK5RAP2 plays a critical role in centriole duplication, PCM cohesion, and microtubule nucleation [20,30].

Truncating mutations in *STARD9* and *CDK5RAP2* have been linked to autosomal recessive microcephaly. For example, a homozygous nonsense mutation within the START domain of *STARD9* was reported in a patient with microcephaly and dwarfism [31]. Likewise, *CDK5RAP2* mutations that disrupt its centrosomal localisation domain are also associated with primary microcephaly, with or without dwarfism [32]. The similarity of phenotypes caused by mutations in each gene individually provides genetic evidence that these genes participate in the same biological process(es). Although these genes have been studied primarily in the context of neurodevelopment, their roles in other organs, including the testes, remain largely unexplored.

Recent studies have begun to uncover a role for *CDK5RAP2* in testis development. In a mouse model, homozygous knockout of *Cdk5rap2* led to underdeveloped testes and failed Sertoli cell polarisation [19]. In humans, a homozygous missense variant in *CDK5RAP2* was associated with non-obstructive azoospermia [33], supporting its role in testicular function. Notably, *STARD9* encodes a protein that belongs to the START domain family, members of which (such as STAR and STARD8) have been implicated in 46,XY DSD, including 46,XY gonadal dysgenesis [12,15,34]. These findings suggest that STARD9, like other START-domain proteins, may have a previously unrecognised role in testis development and function.

We demonstrated that *STARD9* exhibits sexually dimorphic expression, with the highest levels observed in testis-specific Sertoli cells during both human and mouse embryogenesis. This conserved expression pattern supports a role for *STARD9* in Sertoli cell development and, consequently, in the pathogenesis of 46,XY gonadal dysgenesis. In contrast, *CDK5RAP2* shows a more ubiquitous expression profile; however, its expression is higher in Sertoli cells than in female supporting cells. Together, these data support that STARD9 and CDK5RAP2 may interact in Sertoli cells, contributing to their development and function.

In our patient, the *STARD9* variants were compound heterozygous. The variant inherited from the heterozygous father (p.Ser1862_Thr1863del) results in the deletion of two amino acids that are conserved positions among mammals. Because prediction algorithms do not evaluate in-frame deletions, the pathogenicity of this variant cannot be assessed using these tools. This variant has an MAF of 0.001325. The *STARD9* variant inherited from the heterozygous mother (p.Arg1172Cys) leads to the substitution of a non-conserved amino acid and is therefore predicted to be benign by multiple in silico tools (CADD: benign; PolyPhen: benign; SIFT: tolerated). This variant is more common in the population than the paternal variant, with a MAF of 0.009863. Together, these data suggest that the p.Ser1862_Thr1863del variant may have more substantial functional consequences than the p.Arg1172Cys variant. However, the presence of the p.Ser1862_Thr1863del variant in a healthy heterozygous father indicates that the monoallelic form is not pathogenic. The presence of *STARD9* variants on both alleles in the patient means loss of the wild-type protein and could suggest the potential pathogenicity of the compound heterozygous state. In turn, the *CDK5RAP2* p.Tyr668Cys variant occurred in the patient in the heterozygous state and was inherited from the heterozygous mother. This variant is very rare in the population (MAF = 0.0003284). Although prediction programmes classify this substitution as benign (CADD: benign; PolyPhen: benign; SIFT: tolerated), it remains possible that, in combination with the *STARD9* variants, it may contribute to the pathogenic phenotype.

Structural analyses suggest that variants identified in our 46,XY CGD patient may disrupt STARD9 and CDK5RAP2 interactions and function. We found that both STARD9 variants are located in IDRs, which are often involved in post-translational modifications (PTMs) [35,36] and protein–protein interactions [37]. While specific PTMs of STARD9 remain uncharacterised, Ser1862 and Thr1863 may be phosphorylation targets, and Arg1172 may be subject to methylation, alterations that could affect protein interactions and function. In turn, the CDK5RAP2 variant is located within an α-helix. This substitution could disrupt local secondary structure and protein functionality [28,38,39]. Both Arg1172 in STARD9 and Tyr668 in CDK5RAP2 are weakly conserved, which may reflect localisation in regions with lower structural constraints, known to evolve more rapidly [36,40]. Taken together, our structural analysis suggests that the identified variants in STARD9 and CDK5RAP2 may impair their individual and/or combined function(s).

To sum up, the expression of *STARD9* and *CDK5RAP2* in Sertoli cells of embryonic gonads implicates these genes in early testicular development. We propose that coexisting genetic variants in *STARD9* and *CDK5RAP2* may act synergistically to disrupt Sertoli cell development and function, thereby contributing to the pathogenesis of 46,XY gonadal dysgenesis. To our knowledge, this is the first study to implicate *STARD9* and *CDK5RAP2* in 46,XY gonadal dysgenesis.

## 4. Materials and Methods

### 4.1. Compliance with Ethical Standards

The study was conducted in accordance with the Declaration of Helsinki and approved by the Committee on Bioethics of the Institute of Molecular Biology and Genetics of National Academy of Sciences of Ukraine (protocol code No.2, date of approval 30 April 2013).

### 4.2. The Patient

The patient (UKR21) was born after the first normal pregnancy from healthy non-consanguineous parents. The birth weight was 3500 g, and the length was 55 cm. At birth, the child was registered as a female. At 17 years of age, the patient visited a gynaecologist due to an absence of secondary sexual characteristics.

### 4.3. Hormonal Analysis

Blood for hormonal analysis was sampled at 8:00–9:00 am. Levels of T, LH, FSH, E2 and P4 in serum were quantified using electrochemiluminescence immunoassay technology on the Cobas E411 analyser (Roche Diagnostics Ltd., Rotkreuz, Switzerland). The assays were performed using the Elecsys Testosterone II, LH, FSH, E2 III, and Progesterone III kits, following the manufacturer’s instructions (Roche Diagnostics GmbH, Mannheim, Germany). The results were compared to the following reference values for women: T 0.29–1.67 nmol/L; LH (follicular phase) 2.4–12.6 IU/L; FSH (follicular phase) 3.5–12.5 IU/L; E2 (follicular phase) 19.1–135 pmol/L; P4 (follicular phase) 0.159–1.03 nmol/L.

### 4.4. Cytogenetic Studies

Karyotyping was performed according to a standard procedure using the technique of differential staining of G-banding chromosomes (GTG-banding). Cytogenetic studies were performed on peripheral blood lymphocytes (30 metaphase plates) using a Nikon Eclipse Ci microscope (Nikon, Minato, Japan). FISH analysis with Locus-Specific Identifier (LSI) and Centromere Enumeration (CEP) Probes (Yp11.3-*SRY*; Yp11.1-q11.1-*DYZ3*; Yq12-*DYZ1*; CEP-*DXZ1*) was performed at 200 interphase nuclei using LUCIA Cytogenetics 3.1 Software (Praha, Czech Republic) according to Cytogenetic Guidelines and Quality Assurance by European Cytogenetics Association (Abbott Molecular, Libertyville, IL, USA).

### 4.5. Histologic Studies

Gonad samples were collected after gonadectomy. The specimens were fixed in neutral buffered formalin for 72 h, then dehydrated by three changes of 99% isopropanol, cleared in xylene, and embedded in paraffin. Morphological analysis was performed on 5-μm sections that were routinely stained with Ehrlich haematoxylin and eosin. Samples were analysed with light microscopy under ×40–×100 magnification.

### 4.6. WES

Genomic DNA (gDNA) from the blood samples of the patient and her parents was isolated by using the QIAmp DNA Kit (Qiagen, Hilden, Germany). Exome capture was performed on the DNA samples using the SureSelectXT Target Enrichment system for Illumina version B.2 (Agilent Inc^®^, Santa Clara, CA, USA). Paired-end libraries were prepared using TruSeq SBS Kit v3 (Illumina, San Diego, CA, USA) and sequenced on an Illumina HiSeq 4000 system (Illumina, San Diego, CA, USA). Raw sequencing was transformed into .fastq files using the CASAVA v1.8.1 software (Illumina, San Diego, CA, USA) and processed with DRAGEN Germline Pipeline v 2.3 (Edico Genome, San Diego, CA, USA), which leverages Genome Analysis Tool Kit (GATK) developed by the Broad Institute, Cambridge, MA, USA (https://gatk.broadinstitute.org/hc/en-us/articles/360045944831, accessed on 18 January 2018) and is harboured at Illumina’s cloud-based resource BaseSpace.

For the proband and her parents, 100 to 150 million reads were processed, adapter-trimmed, duplicate-marked, and aligned against the GRCh37/hg19 assembly of the human genome using the Smith–Waterman scoring algorithm. At the variant calling stage, the following filters were applied: variant confidence/quality by depth > 2.0, mapping quality > 30.0, phred-scaled *p*-value for strand bias < 60.0, mapping quality RankSum > 12.5, and ReadPos-RankSum > 8.0. Variants were annotated and analysed in Variant Interpreter (Illumina, San Diego, CA, USA) and VarSeq (Golden Helix, Boseman, MT, USA). Further filtering was performed by the variant quality > 500, genotyping quality > 80, read depth > 30, proportion of reads bearing the minor allele > 0.2, and MAF < 0.01.

### 4.7. Validation of the STARD9 and CDK5RAP2 Variants by Sanger Sequencing

The status of the *STARD9* (NM_020759.3):c.3514C>T (p.Arg1172Cys), the *STARD9* (NM_020759.3):c.5585_5590del (p.Ser1862_Thr1863del), and the *CDK5RAP2* (NM_018249.6):c.2003A>G (p.Tyr668Cys) variants in the patient and her parents was confirmed by Sanger sequencing. DNA fragments, each containing one of the above variants, were amplified using the appropriate gDNA templates and specific primers (forward primer TTTCCCAGAGCCAGAGAACT and reverse primer CCCAGTTCTTCCTCTGCAT for the *STARD9*:c.3514C>T variant; forward primer CCTCATCTCAGCAGGTCACA and reverse primer TCCTCTCGTGCCTCAGATTC for the *STARD9*:c.5585_5590del variant; forward primer CCTGGGAAGCTGAAGTCTCT and reverse primer CGCAAGTCTATCTGGAAACCC for the *CDK5RAP2*:c.2003A>G variant). Amplification was performed using the Advantage^®^ 2 PCR Enzyme System with Advantage 2 PCR Buffer or Advantage 2 PCR SA Buffer (Takara Bio, San Jose, CA, USA) according to the manufacturer’s instructions. Each amplicon was sequenced bidirectionally using the respective primers in separate reactions. Additionally, the *STARD9* fragment containing the c.5585_5590del variant was cloned into the pGEM-T Easy vector and resequenced to obtain readable chromatograms. Sequencing was performed using BigDye™ Terminator v3.1 Cycle Sequencing Kit on a 3730xl DNA Analyser (Applied Biosystems, Waltham, MA, USA). Chromatograms were analysed using SnapGene 4.3.11 software (GSL Biotech LLC, San Diego, CA, USA).

### 4.8. STARD9 and CDK5RAP2 Expression Analysis

To assess *STARD9* and *CDK5RAP2* expression in male and female human embryonic gonads, we used single-cell transcriptomic data from Garcia-Alonso et al. (2022) [25], available at https://www.reproductivecellatlas.org/gonads.html. UMAP representations of male gonadal cells were plotted using Scanpy, with parameters vmin = “p0.01”, vmax = “p99.99”. For line plot visualisation, male and female datasets were concatenated. Plots were generated using the Scanpy dotplot function after combining sex and cell type annotations. Mouse data processed as in Mayere et al., 2022 [26] were provided by the authors. UMAP representations of both male and female mouse gonadal cells and the corresponding line plot were generated as for human data.

### 4.9. Molecular Modelling and MD Simulations

To determine the secondary structure of the wild-type STARD9 fragment containing residues Arg1172 and Ser1862_Thr1863 (which are substituted or deleted, respectively, in the patient), several disorder predictors were used: FoldIndex [41], NORSp [42], GlobPlot [43], and CH-Plot [44]. These predictors rely on the physical and chemical characteristics of amino acids and data from known unstructured proteins. Additionally, IUPred [45], Ucon [46] and FoldUnfold [47] inter-residue contact-based predictors were applied. The three-dimensional structures were constructed using AlphaFold2 [48]. Furthermore, the coarse-grained MD simulation of the wild-type STARD9’s large central segment (1136–1888 aa) containing the Arg1172 and Ser1862_Thr1863 residues flanked by 25–36 residues was analysed using the Gromacs 2018.1 software tool [49], along with the CHARMM36 [44] and SIRAH2.0 [50] force fields. The simulation was continued for 700 ns. A constant system temperature was maintained using a Nosé-Hoover thermostat, with simulations performed at 310 K and 1 bar pressure, controlled by a Parrinello–Rahman barostat. A cut-off radius of 12 Å was applied for both Coulomb (electrostatic) and Lennard–Jones (van der Waals) interactions. The Particle-Mesh Ewald method for long-range interactions was used with periodic boundary conditions in the XYZ directions. Finally, the full-atom MD simulation of two mutated STARD9’s short fragments (1163–1179 aa and 1849–1872 aa), each containing either the p.Arg1172Cys or p.Ser1862_Thr1863del mutation flanked by 7–13 residues, was performed for 500 ns to induce secondary structure ordering. The secondary structure of proteins analysis [51] was applied to assess the propensity of these structures to fold under near-natural conditions, and the results were compared with predicted secondary structure assignments.

The CDK5RAP2 protein was rebuilt using AlphaFold2 server [48]. MD of a single helix (650–684 aa that contains Tyr668 residue mutated in the patient) was performed using the Gromacs 2018.1 software tool [49], along with the CHARMM36 [44,52]. The simulation was continued for 200 ns, under the same conditions that we used for the simulations of STARD9 protein fragments.

### 4.10. Bioinformatic Resources and Tools

The Genome Aggregation Database v4.1.0 (https://gnomad.broadinstitute.org/, accessed on 5 November 2025) was used to check the allele frequency of the variants. The public medical genetics databases ClinVar (https://www.ncbi.nlm.nih.gov/clinvar/, accessed on 5 November 2025) and VarSome (https://varsome.com/, accessed on 5 November 2025) were used to check if the variants were registered in any disease. Single-cell atlases of mouse and human gonadal expression were used to check expression of candidate genes (https://www.unige.ch/medecine/nef/datasets and https://www.reproductivecellatlas.org/gonads.html, respectively, accessed on 1 October 2025).

### 4.11. Accession Numbers

The following accession numbers for transcripts were used: NM_020759.3 for *Homo sapiens STARD9*, NM_018249.6 for *Homo sapiens CDK5RAP2*. The following accession numbers for mammalian STARD9 proteins were used: Q9P2P6 for *Homo sapiens*, G3QVK0 for *Gorilla gorilla*, XP_020952823.1 for *Sus scrofa*, F6W6H9 for *Equus caballus*, and Q80TF6 for *Mus musculus*. The following accession numbers for mammalian CDK5RAP2 proteins were used: Q96SN8 for *Homo sapiens*, G3QD67 for *Gorilla gorilla*, A0A8W4FHP0 for *Sus scrofa*, F7DTT1 for *Equus caballus*, and Q8K389 for *Mus musculus*.

## Data Availability

The original contributions presented in this study are included in the article/Supplementary Materials. Further inquiries can be directed to the corresponding authors.

## Supplementary Materials

The supporting information can be downloaded at https://www.mdpi.com/article/10.3390/ijms262311575/s1.

## Abbreviations

The following abbreviations are used in this manuscript:

46,XY CGD	46,XY complete gonadal dysgenesis
46,XY PGD	46,XY partial gonadal dysgenesis
AS	adrenosympathic cells
CDK5RAP2	CDK5 Regulatory Subunit-Associated Protein 2
CE	coelomic epithelial cells
CHD7	Chromodomain Helicase DNA Binding Protein 7
DSD	disorders/differences of sex development
E2	oestradiol
EC	erythrocytes
EIP	early interstitial progenitors
End	endothelial cells
FLC	foetal Leydig cells
FSH	follicle-stimulating hormone
GC	germ cells
IDR	intrinsically disordered region
IM	invading mesonephric cells
Imm.	immune cells
KISS1R	KISS1 Receptor
LGR5	Leucine Rich Repeat Containing G Protein-Coupled Receptor 5
LH	Luteinizing Hormone
LIP	late interstitial progenitors
MAF	the minor allele frequency
MD	molecular dynamics
Meso.	mesonephric mesenchymal cells
MT	mesonephric tubules
P4	progesterone
PCM	pericentriolar material
Pre-sup.	presupporting cells
PTMs	post-translational modifications
PV	perivascular cells
SE	surface epithelial cells
SLC	supporting-like cells
STARD9	Steroidogenic Acute Regulatory-Related Lipid Transfer Domain 9
START	StAR-related lipid transfer domain
T	testosterone
UMAP	Uniform Manifold Approximation and Projection
WES	whole-exome sequencing
