# Supplementary material for: STARD9 and CDK5RAP2—Novel Candidate Genes for 46,XY Complete Gonadal Dysgenesis
Source: Int J Mol Sci. 2025 Nov 28;26(23):11575. doi: 10.3390/ijms262311575 (PMC12692329; doi:10.3390/ijms262311575)
Supplement: Supplementary file 1 [file ijms-26-11575-s001.zip › ijms-4002128-supplementary.pptx]

## Slide 1
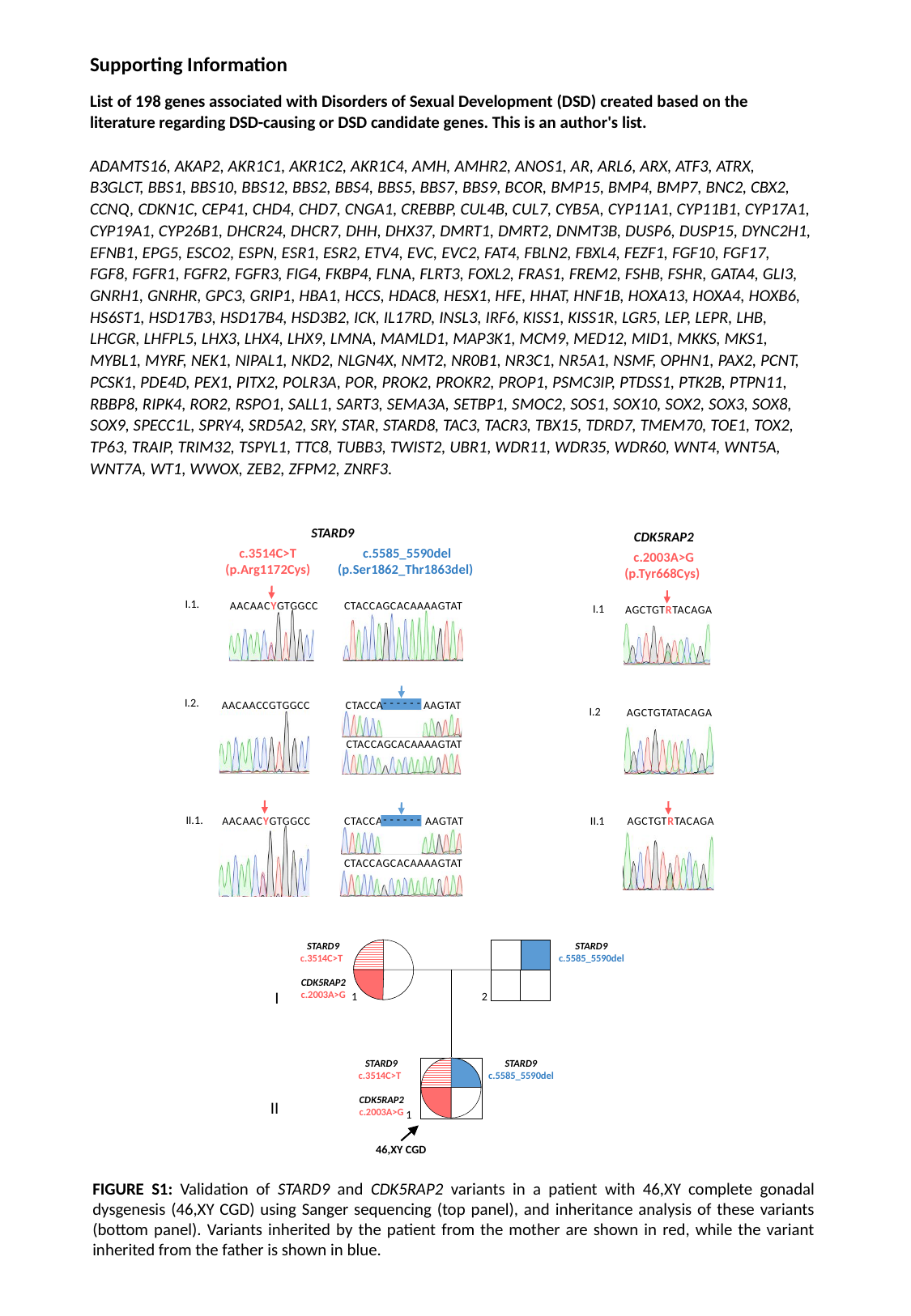

Supporting Information
List of 198 genes associated with Disorders of Sexual Development (DSD) created based on the literature regarding DSD-causing or DSD candidate genes. This is an author's list.
ADAMTS16, AKAP2, AKR1C1, AKR1C2, AKR1C4, AMH, AMHR2, ANOS1, AR, ARL6, ARX, ATF3, ATRX, B3GLCT, BBS1, BBS10, BBS12, BBS2, BBS4, BBS5, BBS7, BBS9, BCOR, BMP15, BMP4, BMP7, BNC2, CBX2, CCNQ, CDKN1C, CEP41, CHD4, CHD7, CNGA1, CREBBP, CUL4B, CUL7, CYB5A, CYP11A1, CYP11B1, CYP17A1, CYP19A1, CYP26B1, DHCR24, DHCR7, DHH, DHX37, DMRT1, DMRT2, DNMT3B, DUSP6, DUSP15, DYNC2H1, EFNB1, EPG5, ESCO2, ESPN, ESR1, ESR2, ETV4, EVC, EVC2, FAT4, FBLN2, FBXL4, FEZF1, FGF10, FGF17, FGF8, FGFR1, FGFR2, FGFR3, FIG4, FKBP4, FLNA, FLRT3, FOXL2, FRAS1, FREM2, FSHB, FSHR, GATA4, GLI3, GNRH1, GNRHR, GPC3, GRIP1, HBA1, HCCS, HDAC8, HESX1, HFE, HHAT, HNF1B, HOXA13, HOXA4, HOXB6, HS6ST1, HSD17B3, HSD17B4, HSD3B2, ICK, IL17RD, INSL3, IRF6, KISS1, KISS1R, LGR5, LEP, LEPR, LHB, LHCGR, LHFPL5, LHX3, LHX4, LHX9, LMNA, MAMLD1, MAP3K1, MCM9, MED12, MID1, MKKS, MKS1, MYBL1, MYRF, NEK1, NIPAL1, NKD2, NLGN4X, NMT2, NR0B1, NR3C1, NR5A1, NSMF, OPHN1, PAX2, PCNT, PCSK1, PDE4D, PEX1, PITX2, POLR3A, POR, PROK2, PROKR2, PROP1, PSMC3IP, PTDSS1, PTK2B, PTPN11, RBBP8, RIPK4, ROR2, RSPO1, SALL1, SART3, SEMA3A, SETBP1, SMOC2, SOS1, SOX10, SOX2, SOX3, SOX8, SOX9, SPECC1L, SPRY4, SRD5A2, SRY, STAR, STARD8, TAC3, TACR3, TBX15, TDRD7, TMEM70, TOE1, TOX2, TP63, TRAIP, TRIM32, TSPYL1, TTC8, TUBB3, TWIST2, UBR1, WDR11, WDR35, WDR60, WNT4, WNT5A, WNT7A, WT1, WWOX, ZEB2, ZFPM2, ZNRF3.
STARD9
c.3514C>T
(p.Arg1172Cys)
 c.5585_5590del
(p.Ser1862_Thr1863del)
I.1.
AACAACYGTGGCC
CTACCAGCACAAAAGTAT
I.2.
- - - - - -
AACAACCGTGGCC
CTACCA AAGTAT
CTACCAGCACAAAAGTAT
- - - - - -
II.1.
AACAACYGTGGCC
CTACCA AAGTAT
CTACCAGCACAAAAGTAT
CDK5RAP2
 c.2003A>G
(p.Tyr668Cys)
I.1
AGCTGTRTACAGA
I.2
AGCTGTATACAGA
AGCTGTRTACAGA
II.1
STARD9
c.3514C>T
STARD9
c.5585_5590del
CDK5RAP2
c.2003A>G
I
2
1
STARD9
c.5585_5590del
STARD9
c.3514C>T
CDK5RAP2
c.2003A>G
II
1
46,XY CGD
FIGURE S1: Validation of STARD9 and CDK5RAP2 variants in a patient with 46,XY complete gonadal dysgenesis (46,XY CGD) using Sanger sequencing (top panel), and inheritance analysis of these variants (bottom panel). Variants inherited by the patient from the mother are shown in red, while the variant inherited from the father is shown in blue.
